# Nutrition-related clinical laboratory indicators in perioperative management of geriatric hip fractures: a narrative review from risk stratification to precision intervention

**DOI:** 10.3389/fnut.2026.1846471

**Published:** 2026-08-10

**Authors:** Xin Xu, Ying Pan, Xuehong Zheng, Junxiang Wang, Chengcheng Zhao, Lin Xiao, Junfei Guo

**Affiliations:** 1 Department of Joint Surgery, Honghui Hospital, Xi'an Jiaotong University, Xi'an, Shaanxi, China; 2 Xi'an Key Laboratory of Pathogenesis and Precision Treatment of Arthritis, Honghui Hospital, Xi'an Jiaotong University, Xi'an, Shaanxi, China; 3 Translational Medicine Center, Honghui Hospital, Xi'an Jiaotong University, Xi'an, Shannxi, China; 4 Nutrition and Dietetics Department, Honghui Hospital, Xi'an Jiaotong University, Xi'an, Shaanxi, China; 5 Department of Orthopedics, Hebei General Hospital, Shijiazhuang, Hebei, China

**Keywords:** albumin (ALB), clinical laboratory indicators, hip fracture, malnutrition, older adults, perioperative management

## Abstract

Geriatric hip fracture represents a major global public health crisis that extends far beyond acute skeletal trauma, reflecting and accelerating systemic physiological decline in older adults. Malnutrition, affecting up to 46% of these patients depending on diagnostic criteria, has been established as a pivotal modifiable determinant of adverse postoperative outcomes including complications, functional decline, and mortality. Against this backdrop, nutrition-related clinical laboratory indicators have gained increasing recognition as objective, quantifiable, and readily accessible tools with potential dual utility in prognostic risk stratification and therapeutic monitoring, yet a comprehensive synthesis of their comparative performance and clinical translation remains lacking. This narrative review therefore aims to systematically synthesize current evidence on the spectrum of nutrition-related laboratory biomarkers ranging from classical protein markers (serum albumin and prealbumin), through inflammatory-nutritional composite indices (Prognostic Nutritional Index, Controlling Nutritional Status score, C-reactive protein/albumin ratio, glucose/albumin ratio, and systemic immune-inflammation index), to emerging proteomic and bone turnover biomarkers, and to critically appraise their predictive value for postoperative complications, functional recovery trajectories, and short- to long-term mortality. We further examine the pathophysiological nexus linking malnutrition, sarcopenia, and osteoporosis, and evaluate evidence-based perioperative nutritional interventions including oral nutritional supplements, enhanced recovery protocols, and multidisciplinary care models. Finally, we delineate future directions encompassing machine learning-driven predictive models, emerging diagnostic frameworks such as the Global Leadership Initiative on Malnutrition criteria, digital nutritional monitoring tools, and the integration of laboratory biomarkers into standardized clinical decision pathways. This review provides a comprehensive framework for clinicians and researchers to advance precision perioperative nutritional management in this vulnerable population.

## 1 Introduction

Geriatric hip fracture (HF) represents one of the most formidable public health challenges worldwide, functioning not merely as an acute skeletal injury but as a sentinel event that both reflects and accelerates systemic physiological decline. In 2019, an estimated 9.6 million new HFs occurred among individuals aged 55 years and older globally, corresponding to an incidence of 681 per 100,000 population (1). Despite continued advances in surgical techniques and perioperative care, clinical outcomes remain sobering, with 1-year mortality rates persisting between 15 and 30%, and fewer than half of survivors achieve full restoration of pre-fracture functional capacity (1, 2). Consequently, HF has been reconceptualized as a systemic event that simultaneously exposes the deficiency of physiological reserves characteristic of the aging body and triggers a cascade of catabolic processes that compound pre-existing frailty, loss of independence, and substantial socioeconomic burden (3).

In the context of this complex clinical problem, malnutrition has been consistently identified as a critical modifiable risk factor governing patient outcomes. The prevalence of malnutrition among geriatric HF patients is alarmingly high. A systematic review encompassing 44 studies and 26,281 patients reported a malnutrition prevalence of approximately 18.7% when assessed by the Mini Nutritional Assessment (MNA), yet this figure rose to 45.7% when alternative criteria including body mass index (BMI), unexplained weight loss, or serum albumin concentration, were applied (2). A comprehensive assessment of 509 acute HF patients revealed that 81.2% exhibited protein malnutrition (serum total protein < 65 g/L or albumin < 35 g/L), while vitamin D deficiency (< 30 ng/mL) was virtually universal at 93% (4). These data underscore that malnutrition in this population is not merely prevalent but constitutes a near-ubiquitous comorbidity. The high prevalence is linked to the nature of HF itself as a potent physiological stressor that triggers an acute catabolic response characterized by accelerated muscle protein breakdown, systemic inflammatory activation, and elevated C-reactive protein (CRP) levels accompanied by concurrent reductions in serum albumin and insulin-like growth factor 1 (IGF-1) (2, 3, 5).

Malnutrition status is independently associated with elevated complication rates, prolonged hospitalization, impaired and incomplete functional recovery, and significantly increased short- and long-term mortality (2, 6). A 5-year prospective study confirmed that baseline nutritional status independently predicted postoperative complications, discharge disposition, readmission rates, prolonged length of stay (LOS), and 5-year mortality (7). Protein-energy malnutrition was shown to correlate with significantly worse recovery trajectories for basic and instrumental activities of daily living (ADL) as well as ambulatory function (8). Franz et al. (9) further demonstrated that malnutrition risk, assessed using the Short Nutritional Assessment Questionnaire (SNAQ), was associated with a 2.6-fold increase in 6-month mortality. These converging lines of evidence firmly establish malnutrition as a central therapeutic target in perioperative management.

Accordingly, perioperative nutritional management has become a fundamental part of multidisciplinary care for geriatric HF patients. Clinical guidelines and expert consensus statements uniformly advocate for systematic nutritional screening, assessment, and intervention commencing upon admission and extending through surgical recovery and community-based rehabilitation (10, 11). The development of standardized clinical management pathways such as those established through Delphi consensus process has further formalized perioperative nutritional care protocols (12). Kapur et al. (13) conducted a large-scale quality improvement program and demonstrated that completion of nutritional screening (e.g., Malnutrition Universal Screening Tool [MUST]) within 24 h of admission was associated with significantly lower in-hospital mortality.

Given this situation, objective and easily obtainable clinical laboratory indicators have become essential for perioperative nutritional management. Compared with subjective composite assessment tools, laboratory biomarkers provide quantifiable data on protein nutritional status, inflammatory burden, and metabolic state. Serum albumin, as the most widely utilized nutritional biomarker, is inexpensive, universally available, analytically standardized, and has been extensively validated as an independent predictor of surgical site infection, acute kidney injury, and both in-hospital and long-term mortality when its preoperative level is diminished (2, 14, 15). However, single-biomarker approaches also carry important biological and preanalytical limitations that constrain their standalone diagnostic and prognostic utility. In particular, serum albumin possesses a comparatively long biological half-life of approximately 18–21 days, which inherently limits its responsiveness to acute nutritional shifts occurring within the compressed perioperative window. As a negative acute-phase reactant, albumin concentrations can also decline substantially in response to trauma- and surgery-induced systemic inflammation independently of true nutritional depletion, and are highly susceptible to dilutional artifact from the aggressive fluid resuscitation and hemodilution frequently administered upon emergency-department admission together potentially masking, mimicking, or exaggerating hypoalbuminemia at critical clinical decision points. Prealbumin, with its shorter half-life of 2–3 days, provides greater sensitivity to acute nutritional shifts, yet shares a similar vulnerability to inflammatory suppression of hepatic synthesis. These intrinsic biological and preanalytical constraints of single biomarkers have collectively driven the field toward emerging inflammatory-nutritional composite indices such as the C-reactive protein/albumin ratio (CAR), the Prognostic Nutritional Index (PNI), the Controlling Nutritional Status (CONUT) score, and the Geriatric Nutritional Risk Index (GNRI), which may confer superior prognostic performance by simultaneously capturing the synergistic pathological interplay between inflammation and nutritional depletion (16). Therefore, a systematic and evidence-based understanding of nutrition-related laboratory indicators spanning classical single-protein biomarkers and their intrinsic limitations, inflammatory-nutritional composite indices, and emerging novel biomarkers is of paramount importance for achieving precision risk assessment, individualized intervention, and outcome optimization in the perioperative management of geriatric HFs.

To achieve this synthesis, the remainder of this review is organized around a logically sequential framework that mirrors the clinical pathway of perioperative nutritional care in geriatric HF. Section 2 first sets the clinical stage by examining the epidemiological burden of malnutrition, its demographic determinants, and the phenotypic clinical screening frameworks, including the MNA-SF, MUST, NRS-2002, and the Global Leadership Initiative on Malnutrition (GLIM) criteria that anchor bedside risk identification. Section 3 then evaluates the classical single-protein nutritional biomarkers, namely serum albumin, prealbumin, and their composite protein derivatives, that constitute the foundational laboratory tier. Building upon these, Section 4 systematically appraises the expanding family of inflammatory-nutritional composite indices, which have emerged specifically to overcome the intrinsic biological and preanalytical limitations of single biomarkers. Section 5 translates the accumulated biomarker evidence into complication-specific prediction paradigms for postoperative pneumonia, delirium, infections, acute kidney injury, and thrombotic events. Section 6 explores the pathophysiological nexus of osteosarcopenia and its emerging bone-turnover, proteomic, and myokine biomarkers. Section 7 examines evidence-based perioperative nutritional interventions, including oral nutritional supplements, specialized formulations, and ERAS-integrated protocols and their measurable impact on both laboratory indicators and functional recovery trajectories. Finally, Section 8 addresses multidisciplinary integration, clinical translation challenges, and the emerging role of digital and artificial-intelligence-driven decision-support frameworks. The review concludes by critically appraising the methodological limitations of the current evidence base (Section 9) and delineating a prioritized future research agenda toward precision perioperative nutritional management (Section 10).

### 1.1 Search strategy and methodology

To ensure methodological transparency, objectivity, and to minimize potential citation bias, a structured literature search strategy was implemented to guide the evidence synthesis of this narrative review. While keeping the comprehensive narrative scope, our methodology aligns with the Scale for the Assessment of Narrative Review Articles (SANRA) guidelines.

We searched PubMed/MEDLINE, Embase, the Cochrane Library, and Web of Science for English-language articles published up to February 2026, The search employed a combination of Medical Subject Headings terms, Emtree terms, and free-text keywords tailored to each database. Boolean operators (AND/OR) were utilized to combine three primary domains: target population, nutritional/laboratory markers, and clinical outcomes. The comprehensive search string was constructed as follows: ((“hip fracture” OR “femoral neck fracture” OR “intertrochanteric fracture”) AND (“nutrition” OR “malnutrition” OR “hypoalbuminemia” OR “albumin” OR “prealbumin” OR “prognostic nutritional index” OR “CONUT” OR “biomarker” OR “clinical laboratory indicators” OR “inflammatory-nutritional composite indices”) AND (“perioperative management” OR “postoperative complications” OR “mortality” OR “functional recovery” OR “risk stratification” OR “precision intervention”)). Manual reference tracking of retrieved articles and relevant systematic reviews was also performed to identify additional eligible studies. Inclusion criteria focused on clinical trials, prospective/retrospective cohort studies, and meta-analyses assessing geriatric populations undergoing surgical repair for acute HFs. Non-English publications, single-case reports, and abstracts without full-text data were excluded. Evidence synthesis was performed qualitatively by assessing the prognostic validity and clinical effect sizes of individual laboratory indicators.

## 2 Epidemiology and comprehensive nutritional assessment

### 2.1 Prevalence and clinical significance of malnutrition

The epidemiological burden of malnutrition among geriatric HF patients is substantial and well documented. In rehabilitation settings, 73% of geriatric HF patients were found to have malnutrition or malnutrition risk (17), while a study of 218 rehabilitation patients using Mini Nutritional Assessment Short-Form (MNA-SF) found that 26.1% were malnourished and 52.6% were at risk, with malnourished patients exhibiting a three-fold higher in-patient mortality rate (18). In a retrospective analysis of a national cohort of 29,377 geriatric HF patients, of whom 17,651 (60.1%) had preoperative serum albumin available, Bohl et al. (19) revealed that 45.9% of those assessed presented with hypoalbuminemia (albumin < 35 g/L), which was associated with a 52% increase in the adjusted risk of 30-day mortality. This discrepancy between MNA-based and biochemical assessments reflects both the heterogeneity of diagnostic criteria and the multidimensional nature of nutritional compromise in this population.

The clinical significance of malnutrition extends across virtually every outcome domain. It is reported that malnutrition independently predicts higher postoperative complication rates, longer LOS, and greater likelihood of requiring higher-level post-discharge care (20). In terms of functional recovery, pre-fracture nutritional status was identified as a significant independent predictor of functional status at acute-phase discharge as measured by the Functional Independence Measure (FIM) (21), and nutritional improvement during rehabilitation was independently associated with superior ADL recovery at discharge (22).

### 2.2 Demographic variations in nutritional status

Demographic factors exert substantial influence on nutritional profiles in HF patients. Females make up 73.5% of the typical patient demographic, with an average age surpassing 83 years, and demonstrate higher prevalence of both malnutrition and vitamin D deficiency compared with their male counterparts (2, 4). Age is an independent determinant of post-fracture outcome. In a prospective multicentre cohort, patients aged 77–82 and 83–99 years had 4.25- and 3.82-fold higher adjusted 6-month mortality risks, respectively, compared with those aged 50–69 years. In the same model, malnutrition risk remained an independent predictor of 6-month mortality (HR 2.61, 95% CI 1.34–5.06) after adjustment for age, sex, and comorbidity burden (9). Cognitive impairment further compounds nutritional vulnerability with a study of more than 800 HF patients found that 11.3% of cognitively impaired patients (Abbreviated Mental Test Score < 7) were at nutritional risk, compared with 6.5% of those with preserved cognition (23). Importantly, cognitive impairment not only increases malnutrition risk but also reduces adherence to nutritional interventions, creating a particularly recalcitrant clinical challenge (24).

### 2.3 Nutritional screening and assessment tools

Numerous screening and assessment methods have been developed and validated within this population. Among these, the MNA-SF have been most extensively investigated, which is not only effectively identifies patients with malnutrition or malnutrition risk (25) but also demonstrates robust predictive validity for mortality, with a meta-analysis reporting a 3.61-fold mortality risk for patients with low MNA-SF scores (26). Crucially, a prospective head-to-head comparison of MNA-SF, MUST, NRS-2002, and GNRI revealed that only MNA-SF was significantly associated with all functional outcome measures, including motor function at discharge, rehabilitation efficiency, and postoperative gait velocity (27). In addition, MNA-SF has also demonstrated independent predictive value for postoperative delirium, 6-month readmission, and 36-month mortality, outperforming both MUST and NRS-2002 (28, 29).

In addition to these phenotypic screening tools, several laboratory-derived composite indices, most notably the GNRI, the PNI, and the CONUT score are also frequently applied to nutritional risk assessment in geriatric HF (30–33). However, because these indices are intrinsically constructed by integrating biochemical and inflammatory-immunological parameters rather than purely phenotypic screening variables, they are more appropriately conceptualized as inflammatory-nutritional composite biomarkers. Their formulations, optimal thresholds, comparative prognostic performance, and clinical utility across specific perioperative endpoints are therefore systematically appraised within the dedicated framework of Section 4.

The GLIM criteria represent an important diagnostic advancement. By combining phenotypic criteria (weight loss, low BMI) with etiologic criteria (reduced food intake, inflammation), GLIM provides a more comprehensive assessment framework. Wu et al. (34) demonstrated that GLIM achieved a higher AUC (0.862) for predicting 1-year hip joint function recovery compared with NRS-2002 (0.812) and MNA-SF (0.763). Table 1 provides a comprehensive comparison of the diagnostic characteristics and predictive performance of validated nutritional assessment tools in geriatric HF patients.

**Table 1:** Nutritional assessment tools in geriatric hip fracture patients: diagnostic characteristics and predictive performance.

Tool	Study design and level of evidence (LoE)	Representative sample size/pool	Components	Outcome assessed	Key effect estimates and predictive value	References
MNA/MNA-SF	Meta-analyses and cohorts (LoE I–III)	Pool: >26,281 patients	Dietary intake, anthropometrics, global assessment, self-perception	Short/long-term mortality; functional capacity	OR 3.61 for low MNA-SF score; high predictive validity for 36-month mortality	(25–29)
GNRI	Cohorts and meta-analyses (LoE I–III)	Single cohorts to large meta-pools	(1.489 × albumin) + (41.7 × weight/ideal weight)	30-day and long-term mortality; gait velocity	Independent predictor of mortality; combined with CLR yields 90% accuracy for 3-month survival	(26, 27, 30–32)
PNI	Retrospective cohorts (LoE III)	Cohorts ranging from hundreds to thousands	Albumin (g/L) + 5 × lymphocyte count ( × 10?/L)	3-year mortality; POD; ICU admission	Highest quartile: 74% lower 3-year mortality (HR 0.26)	(77–83)
CONUT	Retrospective cohorts (LoE III)	Multi-center cohorts	Albumin, total cholesterol, lymphocyte count (scored 0–12)	Postoperative complications; 180-day walking independence	Independent predictor of postoperative complications; Moderate-to-severe malnutrition: 1.42-fold increase in risk of losing walking independence	(33, 84, 85)
GLIM criteria	Retrospective cohorts (LoE III)	Specialized clinical validation cohorts	Phenotypic (weight loss, low BMI) + etiologic (reduced food intake, inflammation)	1-year motor function and hip joint recovery trajectory	Motor recovery prediction: AUC 0.862 (outperforming NRS-2002 [AUC 0.812] and MNA-SF [AUC 0.763])	(34)
HALP score	Retrospective cohorts (LoE III)	Cohorts up to 1,707 patients	Hemoglobin × albumin × lymphocyte/platelet	90-day and overall survival rates	Tertile 3 vs. 1: HR 0.598 for 90-day mortality; HR 0.618 for overall mortality	(61)

LoE, level of evidence; MNA, mini nutritional assessment; MNA-SF, mini nutritional assessment short-form; GNRI, geriatric nutritional risk index; PNI, prognostic nutritional index; CONUT, controlling nutritional status; GLIM, global leadership initiative on malnutrition; HALP, hemoglobin, albumin, lymphocyte, and platelet; CLR, C-reactive protein-to-lymphocyte ratio; POD, postoperative delirium; ADL, activities of daily living; HR, hazard ratio; OR, odds ratio; AUC, area under the receiver operating characteristic curve; BMI, body mass index.

### 2.4 The association between malnutrition, frailty, and sarcopenia

The vulnerability of HF patients is underpinned by the pathophysiological connection between malnutrition, frailty, and sarcopenia. Li et al. (35) found that 49.08% were frail, with malnutrition being a significant contributing factor, and MNA scores have been shown to predict frailty status effectively, serving as a low-cost screening tool (36). Sarcopenia is highly prevalent in this population, affecting approximately 37% of HF patients and at rates significantly higher than in age-matched osteoarthritis patients (37, 38). Importantly, HF patients with sarcopenia demonstrate significantly lower albumin, prealbumin, and hemoglobin levels compared with non-sarcopenic counterparts (38). This combination creates a loop that reinforces itself: malnutrition drives muscle wasting and bone loss; sarcopenia increases fall risk and fracture susceptibility; and frailty diminishes physiological reserve, impairing recovery and predisposing to further nutritional decline. Integrating nutritional assessment with frailty and sarcopenia evaluation, for instance, combining CONUT scores with ADL assessments has been shown to optimize prognostic performance for 1-year mortality (39). However, clinicians must be cognizant of multicollinearity among nutritional, inflammatory, and frailty indicators when constructing predictive models, as failure to account for this may lead to erroneous identification of independent risk factors (40).

## 3 Key protein nutritional indicators: prognostic value and clinical interpretation

### 3.1 Serum albumin

Serum albumin, as the primary prognostic biomarker, is the most extensively examined and clinically utilized nutritional biomarker in geriatric HF treatment, due to its convenient detection and low cost. An overwhelming body of high-quality evidence establishes preoperative hypoalbuminemia as an independent risk factor for both short-term and long-term mortality. In the analytic cohort of 17,651 patients with available albumin data, Bohl et al. (19) demonstrated that patients with hypoalbuminemia (< 35 g/L) had significantly higher 30-day mortality (9.94 vs. 5.53%; adjusted relative risk 1.52, 95% CI 1.37–1.70). Specifically, compared with patients with albumin < 35 g/L, those with levels of 35–40 g/L and ≥40 g/L had 29 and 38% lower long-term mortality risks, respectively (41). Furthermore, this predictive capacity persists at 30 days postoperatively and is independent of age, sex, BMI, and comorbidity burden (42, 43).

Beyond mortality, hypoalbuminemia is strongly and independently associated with multiple perioperative complications. The most robust association is with postoperative pneumonia (POP) that preoperative albumin < 35 g/L increases POP risk with odds ratios ranging from 5.19 to 6.18 (44, 45), while early postoperative albumin below 30 g/L independently predicts POP (46). Besides, low albumin is also independently associated with postoperative acute kidney injury (47, 48), postoperative delirium (49), urinary tract infection (50), preoperative deep vein thrombosis (51), and even cannulated screw fixation failure in femoral neck fractures (52).

The impact of hypoalbuminemia on functional recovery is equally consequential. Preoperative albumin ≤ 35 g/L independently predicts inferior hip function scores, mobility, and quality of life at 6 months (53), as well as inability to walk independently at 6 weeks postoperatively (54). Low albumin also portends a higher 30-day readmission risk (55, 56). Serum albumin acts as a complete indicator of protein reserves, inflammation levels, and overall physiological strength, reflecting the body's ability to endure surgical stress and achieve functional restoration.

### 3.2 Prealbumin

Prealbumin, with its shorter half-life of 2–3 days and smaller body pool relative to albumin with half-life ~20 days, offers greater sensitivity for detecting short-term nutritional changes and protein synthesis rates. In a cohort study of 2,387 patients, Chen et al. revealed a non-linear association between admission prealbumin and long-term mortality, with a threshold effect at 162.2 mg/L. Below this inflection point, each 10 mg/L increase in prealbumin corresponded to a 7% reduction in mortality risk, whereas above this level, the association was no longer significant (57). This finding positions prealbumin as a particularly informative biomarker in the lower concentration range, precisely where clinical concern is greatest. Low prealbumin has been confirmed as an independent risk factor for both 1-year survival and independent walking ability, with AUC values approximating 0.70 for predicting 6-month and 1-year survival (58). Additionally, low prealbumin has also been found independently predicts 30-day readmission (56).

However, it is essential to recognize that, like albumin, prealbumin is a negative acute-phase reactant whose levels decline during systemic inflammation. Gunnarsson et al. (5) concluded that prealbumin decreased significantly postoperatively in both high-energy and standard nutritional intervention groups, underscoring that acute inflammatory suppression of hepatic synthesis may obscure the nutritional signal.

### 3.3 Composite albumin-based and protein indices

Recognizing the complementary strengths of albumin and prealbumin, researchers have developed composite indices to enhance predictive performance. The albumin-hemoglobin index (AHI), which integrates protein nutritional status with oxygen-carrying capacity, demonstrated AUC values reaching 0.75 for predicting 1-year mortality, comparable to the red cell distribution width/albumin ratio (59). The prealbumin-adjusted PNI (PAPNI), constructed by substituting prealbumin for albumin in the traditional PNI formula, has shown superior accuracy in predicting 1-year mortality and free walking ability compared with conventional PNI and either prealbumin or lymphocyte count alone (60). The Hemoglobin, Albumin, Lymphocyte, and Platelet (HALP) score represents another novel composite. In a study of 1,707 patients, Wang et al. revealed that higher HALP scores were associated with significantly lower 90-day and overall mortality (61).

### 3.4 Clinical interpretation and considerations

Rigorous clinical interpretation of protein nutritional markers demands recognition of their sensitivity to multiple physiological and pathological influences. Albumin and prealbumin levels are jointly regulated by nutritional intake, hepatic synthetic capacity, and the magnitude of systemic inflammation (62). The immediate inflammatory reaction triggered by fractures and surgery leads to a physiological drop in these proteins shortly after the operation, which doesn't necessarily indicate a decline in nutrition (5, 63). Accordingly, dynamic trend monitoring may be more clinically informative than single-point measurements, with sustained postoperative hypoalbuminemia carrying greater prognostic significance than an isolated preoperative low value (47, 64). Furthermore, hypoalbuminemia is frequently accompanied by vitamin D deficiency, which may synergistically impair skeletal health and recovery (65, 66). Clinicians should therefore interpret protein markers within the broader context of inflammatory status, hepatic function, and micronutrient sufficiency to avoid both overdiagnosis and undertreatment. The prognostic associations of serum albumin and prealbumin with specific perioperative outcomes are systematically summarized in Table 2.

**Table 2:** Prognostic associations of serum albumin and prealbumin with perioperative outcomes in geriatric hip fracture patients.

Biomarker	Clinical outcome assessed	Threshold/ Measure	Effect size	Key findings	References
Serum albumin	In-hospital mortality	< 35 g/L	52% increased adjusted mortality risk (9.94 vs. 5.53%)	Clear dose-response gradient	(19)
	Long-term mortality	35–40 vs. < 35 g/L	29% mortality risk reduction for 35–40 g/L; 38% reduction for ≥40 g/L	Independent of age, sex, BMI, comorbidity burden	(41–43)
	Postoperative pneumonia	< 35 g/L (preop.); < 30 g/L (early postop.)	OR 5.187–6.18 (preop.); bidirectional relationship	Dose-response pattern; impaired immune function and low PaO_2_	(44–46)
	Postoperative delirium	< 35 g/L	OR 2.99–3.97; 11% risk increase per 1 g/L decrease	Dose-response gradient confirmed	(49)
	Acute kidney injury	Early postoperative low albumin	OR 1.80 (meta-analysis)	Independent of preoperative creatinine	(47, 48, 99)
	Functional recovery	≤ 35 g/L	Inferior hip function, mobility, QoL at 6 months	Risk factor for inability to walk independently at 6 weeks	(53, 54)
	30-day readmission	Low admission albumin	Independent predictor	Also predicts DVT, UTI, fixation failure	(50–52, 55, 56)
Prealbumin	Long-term mortality	Inflection point at 162.2 mg/L	7% mortality reduction per 10 mg/L increase (below inflection)	Non-linear association	(57)
	1-year survival and walking ability	Low prealbumin	AUC approximately 0.70 for 6-month and 1-year survival	Independent risk factor for walking disability	(58)
	30-day readmission	Low admission level	Independent predictor	More sensitive to short-term changes (half-life 2–3 days)	(56)

preop., preoperative; postop., postoperative; OR, odds ratio; HR, hazard ratio; AUC, area under the receiver operating characteristic curve; PaO_2_, arterial oxygen tension; DVT, deep vein thrombosis; UTI, urinary tract infection; QoL, quality of life; BMI, body mass index.

All effect sizes represent adjusted estimates unless otherwise indicated.

## 4 Inflammatory-nutritional composite indices: a multilayered predictive framework

### 4.1 Simple hematological and inflammatory ratios

Due to the acute stress and inflammation from HF, relying solely on nutritional indicators is not enough for a comprehensive risk assessment. Simple ratios derived from routine laboratory panels represent the first tier of inflammatory-nutritional integration. Red cell distribution width (RDW), reflecting erythrocyte volume heterogeneity associated with inflammation and micronutrient deficiency, has been validated as a significant predictor of postoperative mortality. Admission RDW >14.5% independently predicts short- and long-term mortality (67), and a meta-analysis of 10 studies confirmed that elevated preoperative RDW is associated with increased mortality at 30 days, 3 months, 6 months, and 1 year (68). The neutrophil-to-lymphocyte ratio (NLR), a readily calculable marker of systemic inflammation, correlates with postoperative complications, though its standalone predictive power is limited (69). In addition, the platelet-to-lymphocyte ratio (PLR) has been also demonstrated independently associated with 90-day mortality (70).

### 4.2 Metabolic stress–nutrition ratios

Ratios integrating metabolic stress markers with nutritional reserves provide more nuanced risk stratification. The glucose-to-albumin ratio (GAR) captures the dual insult of stress hyperglycemia and protein depletion. Preoperative GAR has been identified as an independent predictor of POP, with predictive performance superior to many conventional indicators (71), as well as independently predicts postoperative delirium, with each 0.1-unit increase in GAR conferring a 1.6-fold risk elevation (72). The CRP-to-albumin ratio (CAR), integrating acute-phase inflammation with nutritional status, has been associated with significantly increased 1-year mortality in multiple studies (73, 74). The albumin-to-globulin ratio (AGR) demonstrates a non-linear association with POP, with a significant inverse relationship below a threshold of 1.33 (75). The D-dimer-to-albumin ratio (DAR), when combined with NLR, offers promising predictive value for preoperative lower extremity deep vein thrombosis (76).

### 4.3 Comprehensive scoring systems

In recent years, multi-parameter scoring systems represent the most sophisticated tier of inflammatory-nutritional assessment. The PNI, calculated as serum albumin (g/L) + 5 × total lymphocyte count ( × 10^9^/L), has accumulated the most robust evidence base. A *post-hoc* analysis study demonstrated that patients in the highest PNI quartile had a 74% lower 3-year mortality compared with the lowest quartile (77). Lower PNI independently predicts postoperative complications, 2-year all-cause mortality, postoperative delirium, postoperative ICU admission, and moderate-to-severe ADL dependence at 2 years (78–83).

The CONUT score, integrating serum albumin, total cholesterol, and total lymphocyte count, independently predicts postoperative complications (84) and, when indicating moderate-to-severe malnutrition, is associated with a 1.42-fold increase in the risk of losing walking independence at 180 days postoperatively (85). The Systemic Immune-Inflammation Index (SII), derived from neutrophil, platelet, and lymphocyte counts, has been associated with increased 2-year all-cause mortality and identified as a potential biomarker for postoperative delirium in intertrochanteric fracture patients (86, 87).

### 4.4 Machine learning–based predictive model integration

The evolution from single indicators to machine learning–driven multivariate model represents the frontier of prognostic assessment. Machine learning algorithms have identified age, blood glucose, RDW, and mean corpuscular hemoglobin concentration as the most important features for predicting 1-year mortality (88). Random forest and other algorithms have been successfully applied to construct models predicting preoperative cerebral infarction based on prealbumin, fibrinogen, and globulin (89). For postoperative delirium prediction, CatBoost and other advanced algorithms integrating preoperative albumin, glucose, creatinine, and nutritional status have achieved superior performance (90). Furthermore, incorporating serum albumin into the Nottingham Hip Fracture Score improved 30-day mortality discrimination (C-statistic from 0.68 to 0.74), whereas inflammatory markers such as CRP and NLR did not yield comparable improvement (91). A comprehensive prognostic model incorporating nine predictors including age and albumin achieved a C-statistic of 0.814 for 1-year survival prediction (92). These advances highlight the promise of data-driven approaches in extracting maximal prognostic information from routine laboratory parameters.

Despite their theoretical promise, the transition of machine learning models from retrospective development to routine orthogeriatric practice remains severely constrained by critical methodological hurdles. The vast majority of current diagnostic and prognostic algorithms are trained on relatively small sample sizes or single-center datasets, which introduces a substantial risk of algorithmic overfitting and over-optimistic performance metrics. Furthermore, there is a profound lack of rigorous external validation across geographically and ethnically diverse patient populations to confirm generalizability. Because of these limitations, combined with the logistical complexity of integrating complex algorithmic software into existing electronic health record architecture, actual real-time clinical implementation of these models remains virtually non-existent at the bedside. Consequently, machine learning frameworks must currently be viewed as emerging investigational tools rather than mature clinical solutions ready for routine, standardized deployment in perioperative management.

**Figure 1:**
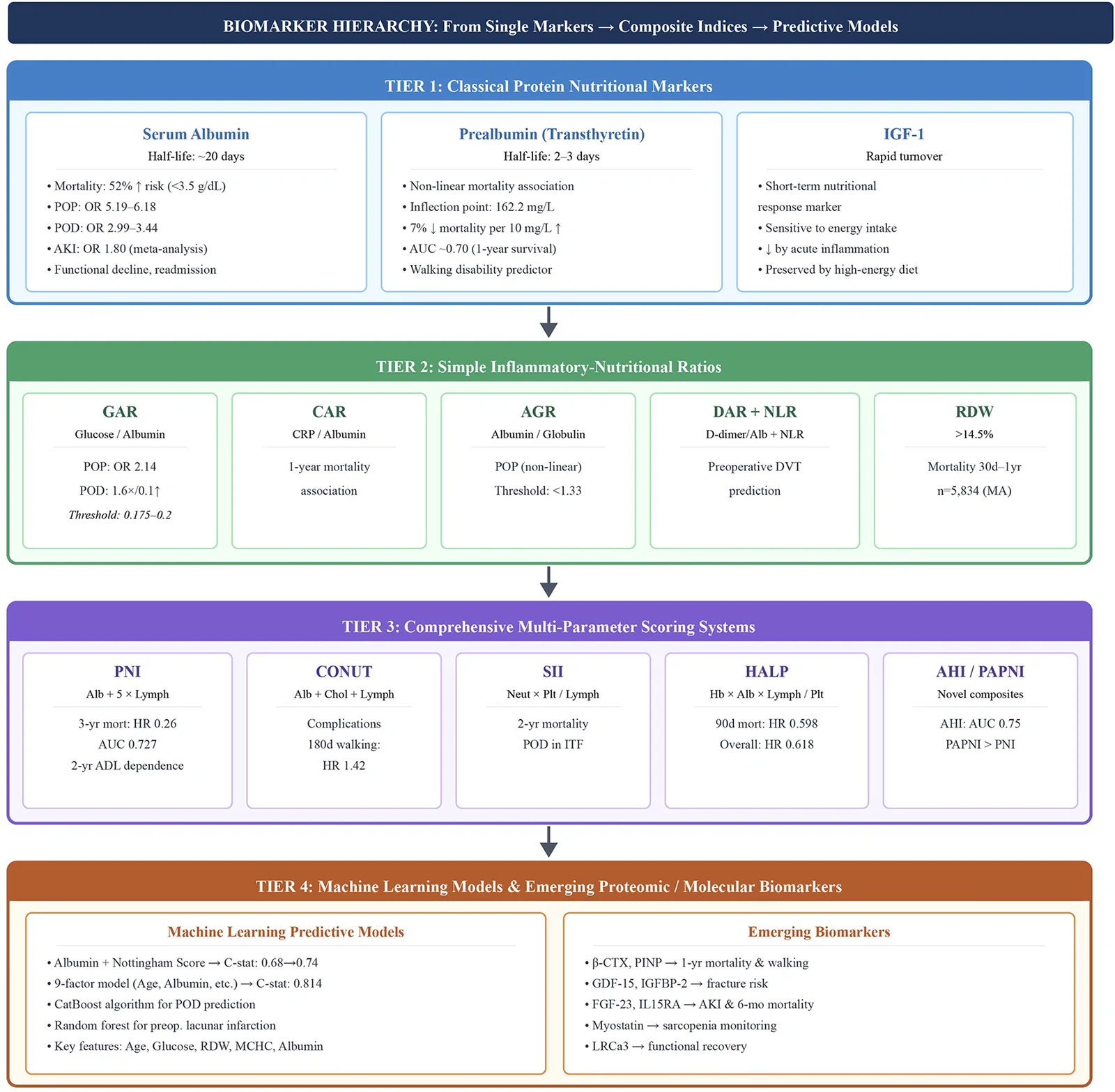
Hierarchical spectrum of nutrition-related laboratory biomarkers in geriatric hip fracture, organized by increasing complexity across four tiers. Tier 1 encompasses classical protein nutritional markers (albumin, prealbumin, IGF-1) with established prognostic associations. Tier 2 includes simple inflammatory-nutritional ratios derived from routine laboratory panels. Tier 3 comprises comprehensive multi-parameter scoring systems integrating nutritional and inflammatory data. Tier 4 represents the frontier of machine learning–integrated predictive models and emerging proteomic/molecular biomarkers. Key predictive associations and effect sizes are indicated for each biomarker. This framework guides clinicians in selecting appropriate indicators based on clinical context, prognostic needs, and available resources. POP, postoperative pneumonia; POD, postoperative delirium; AKI, acute kidney injury; IGF-1, insulin-like growth factor 1; GAR, glucose-to-albumin ratio; CAR, CRP-to-albumin ratio; AGR, albumin-to-globulin ratio; DAR, D-dimer-to-albumin ratio; CLR, C-reactive protein-to-lymphocyte ratio; RDW, red cell distribution width; PNI, Prognostic Nutritional Index; CONUT, Controlling Nutritional Status; SII, Systemic Immune-Inflammation Index; HALP, Hemoglobin, Albumin, Lymphocyte, and Platelet; AHI, albumin-hemoglobin index; PAPNI, prealbumin-adjusted PNI; ADL, activities of daily living; β-CTX, β-C-terminal telopeptide of type I collagen; PINP, N-terminal propeptide of type I procollagen; GDF-15, growth differentiation factor 15; IGFBP-2, insulin-like growth factor binding protein 2; FGF-23, fibroblast growth factor 23; IL15RA, interleukin-15 receptor alpha; LRCa3, lymphocyte ratio-calcium index.

Table 3 systematically summarizes the definitions, optimal thresholds, and predictive performance of inflammatory-nutritional composite indices evaluated in geriatric HF populations. Figure 1 presents a hierarchical overview of the spectrum of nutrition-related laboratory biomarkers discussed in Sections 3 and 4, organized across four tiers of increasing complexity from classical protein markers to machine learning–integrated predictive models and emerging proteomic biomarkers, providing a framework to guide clinicians in selecting appropriate indicators based on clinical context and available resources.

**Table 3:** Inflammatory-nutritional composite indices: definitions, optimal thresholds, and predictive performance for perioperative outcomes.

Index	Formula/ Components	Category	Outcome predicted	Threshold	Effect size/Performance	References
RDW	Red cell distribution width (%)	Hematological	Short- and long-term mortality	>14.5%	Significant predictor at 30 days, 3, 6, and 12 months (meta-analysis)	(67, 68)
NLR	Neutrophils/lymphocytes	Inflammatory	Postoperative complications	Variable	Correlated but limited standalone predictive power	(69)
PLR	Platelets/lymphocytes	Inflammatory	90-day mortality	Variable	Independent association	(70)
GAR	Glucose/albumin	Metabolic-nutritional	POP; POD	0.175–0.2	POP: OR 2.14; POD: 1.6-fold increase per 0.1-unit rise	(71, 72)
CAR	CRP/albumin	Inflammatory-nutritional	1-year mortality	Variable	Significantly elevated in non-survivors	(73, 74)
AGR	Albumin/globulin	Nutritional	POP	< 1.33	Non-linear inverse association with POP below threshold	(75)
DAR + NLR	D-dimer/albumin combined with NLR	Coagulation-inflammatory	Preoperative DVT	Combined model	Promising predictive value for lower extremity DVT	(76)
AHI	Albumin × hemoglobin	Nutritional-hematological	1-year mortality	Variable	AUC 0.75; comparable to RAR	(59)
SII	Neutrophils × platelets/lymphocytes	Inflammatory	2-year mortality; POD	Variable	Significant independent association with both endpoints	(86, 87)
LRCa3	Lymphocyte ratio × calcium index	Inflammatory-metabolic	1-year functional recovery	Novel	Emerging predictor of postoperative functional outcomes	(148)

RDW, red cell distribution width; NLR, neutrophil-to-lymphocyte ratio; PLR, platelet-to-lymphocyte ratio; GAR, glucose-to-albumin ratio; CAR, C-reactive protein-to-albumin ratio; AGR, albumin-to-globulin ratio; DAR, D-dimer-to-albumin ratio; AHI, albumin-hemoglobin index; SII, systemic immune-inflammation index; LRCa3, lymphocyte ratio-calcium index; RAR, red cell distribution width-to-albumin ratio; POP, postoperative pneumonia; POD, postoperative delirium; DVT, deep vein thrombosis; AUC, area under the receiver operating characteristic curve; CRP, C-reactive protein.

### 4.5 Comparative synthesis and clinical choice

To optimize bedside clinical decision-making, the extensively discussed biomarkers must be directly compared and synthesized. Currently, serum albumin possesses the strongest and most ubiquitous volume of evidence, establishing it as the absolute gold standard for baseline risk screening. When evaluating predictive performance for mortality, serum albumin and the GNRI demonstrate the highest consistency for both short- and long-term survival trajectories. For predictive accuracy regarding perioperative complications, multi-parameter scoring systems such as the PNI for delirium and intensive care requirement and the CONUT score for surgical site and systemic complications outperform single protein markers by capturing the cross-talk between nutritional depletion and immune-inflammatory activation. Regarding routine clinical practicality and cost-effectiveness, albumin, prealbumin, PNI, CONUT, and GNRI represent the tier-one choices. PNI, GNRI, and CONUT require zero additional institutional cost because they are calculated entirely from routine, universally mandated admission blood panels (albumin, lymphocytes, total cholesterol, hemoglobin, and weight). In contrast, specific metabolic-inflammatory ratios like the GAR excel uniquely in predicting individual complications such as postoperative pneumonia and delirium, but they currently hold secondary recommendations due to a lack of multi-center standardization.

## 5 Laboratory-based prediction of specific perioperative complications

### 5.1 Postoperative pneumonia

Both preoperative and early postoperative hypoalbuminemia are firmly established as independent risk factors for POP, which is a leading cause of mortality in geriatric HF patients. According to previous studies, preoperative albumin below 35 g/L is associated with substantially elevated POP risk, with odds ratios ranging from 5.187 to 6.18 (44, 45). Shin et al. (64) demonstrated that early postoperative albumin nadir < 30 g/L within the first two postoperative days could independently predict POP. The bidirectional relationship between hypoalbuminemia and POP is noteworthy that low albumin predisposes to pneumonia, while pneumonia further depresses albumin levels, creating a vicious cycle (46). Hypoalbuminemia impairs both systemic immune function and pulmonary gas exchange, with low albumin correlating with reduced arterial oxygen tension (PaO_2_) (93). Among composite indices, GAR demonstrates superior predictive performance, with preoperative GAR exceeding 0.175–0.2 conferring an odds ratio of 2.14 for POP (71), while AGR below 1.33 exhibits a significant inverse relationship with POP risk (75). These indicators provide actionable targets for intensified respiratory care and nutritional optimization in high-risk patients, especially for male patients (93, 94).

### 5.2 Postoperative delirium

POD is a multifactorial syndrome in which nutritional and inflammatory dysregulation feature prominently. Preoperative hypoalbuminemia < 35 g/L is among the most consistently identified independent risk factors, with adjusted odds ratios of 2.99–3.97, and the association demonstrates a significant dose-response gradient (49, 95). Elevated GAR, SII, and CRP, and low PNI has been confirmed as independent predictors for POD (72, 80, 87). Clinical prediction models integrating laboratory and clinical variables including age >75 years, stroke history, preoperative hemoglobin ≤ 100 g/L, PaO_2_ ≤ 60 mmHg, and time to surgery >3 days, achieve good sensitivity and specificity (96). Advanced machine learning models such as CatBoost further improve individualized prediction accuracy (90). Besides, reduced thyroid-stimulating hormone has also been identified as a POD risk factor (95, 97). These findings suggest that correcting hypoalbuminemia, controlling glycemia, and modulating inflammation may represent viable delirium prevention strategies.

### 5.3 Infections, acute kidney injury, and thrombotic complications

Moreover, nutritional-inflammatory biomarkers predict a broad spectrum of additional perioperative complications. Yao et al. (50) found preoperative hypoalbuminemia may independently predict urinary tract infection (OR 1.86) with a dose-response pattern. Rutenberg et al. (98) concluded that PNI and protein-energy malnutrition serve as effective screening tools for surgical site infection risk. For acute kidney injury (AKI), early postoperative hypoalbuminemia is an independent risk factor, with a meta-analysis reporting an OR of 1.80 (99). Notably, preoperative inflammatory biomarkers related to renal function such as fibroblast growth factor 23 and interleukin-15 receptor alpha has been demonstrated that elevated levels were associated with postoperative AKI and 6-month mortality, with AKI partially mediating the relationship between these biomarkers and mortality (100). Besides, preoperative hypoalbuminemia is further associated with deep vein thrombosis (101), calf muscular vein thrombosis (102), and internal fixation failure (52). These associations collectively reveal that nutritional depletion, as reflected by low albumin, represents a common pathological nexus linking diverse perioperative complications across organ systems.

## 6 Osteosarcopenia and nutritional interplay: emerging biomarker perspectives

### 6.1 Epidemiology and pathophysiology of osteosarcopenia

In geriatric HF patients, malnutrition, sarcopenia, and osteoporosis do not exist as isolated pathologies but rather form an intimately interconnected triad termed “osteosarcopenia”—a clinical syndrome reflecting synchronous deterioration of the musculoskeletal system. The prevalence of osteosarcopenia ranges from 5 to 37% in community-dwelling older adults to as high as 17.1%−96.3% in HF populations (103). Compared with patients with isolated osteoporosis or sarcopenia, those with osteosarcopenia exhibit worse nutritional status, inferior balance and functional capacity, and significantly higher risks of falls, re-fracture, and earlier mortality.

**Figure 2:**
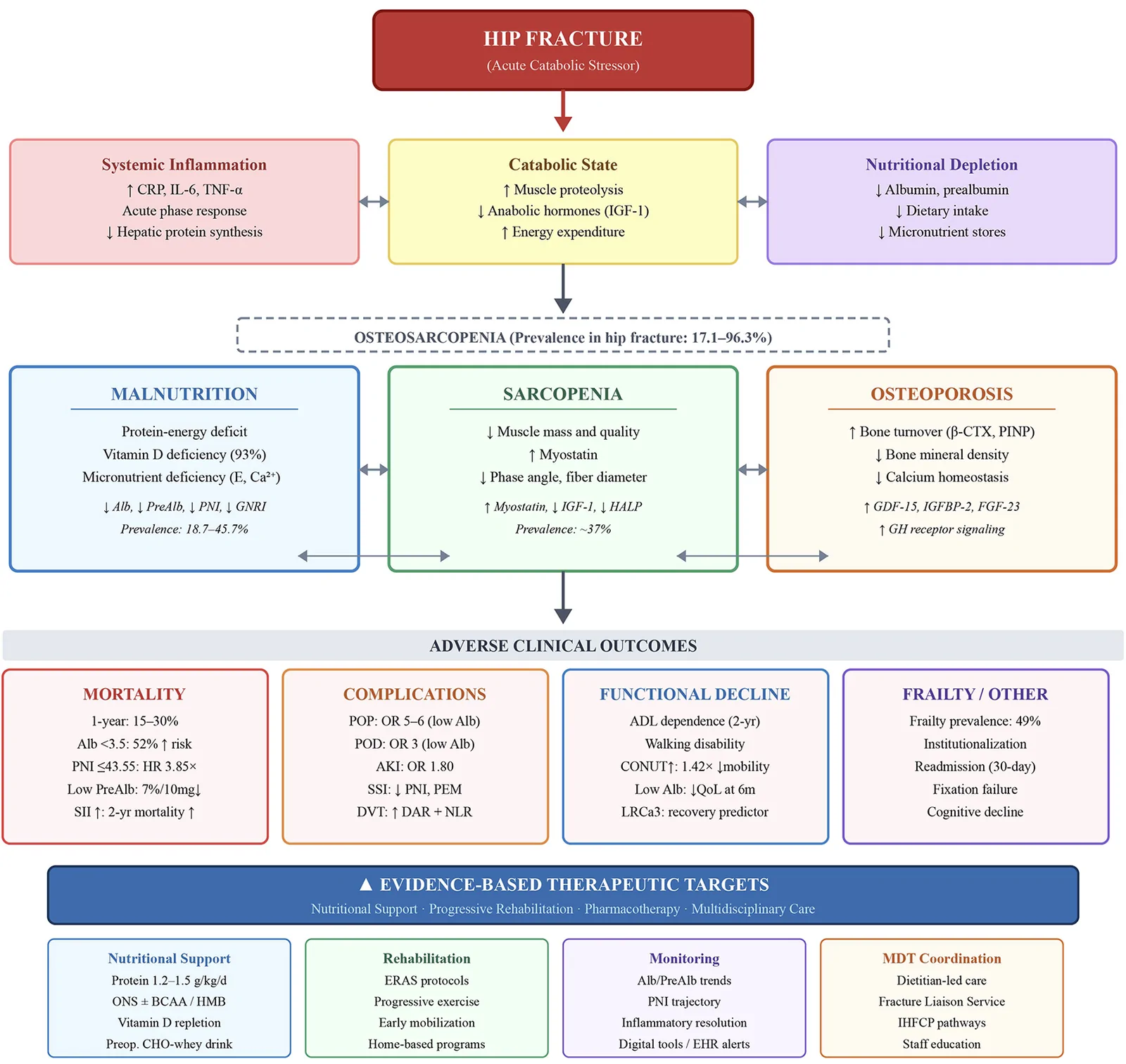
Pathophysiological framework of the malnutrition–inflammation–osteosarcopenia nexus in geriatric hip fracture. The diagram illustrates how the acute catabolic response triggered by hip fracture, characterized by systemic inflammation, enhanced catabolism, and nutritional depletion, drives the interconnected triad of malnutrition, sarcopenia, and osteoporosis (osteosarcopenia). Bidirectional arrows denote mutually reinforcing pathological interactions. Key laboratory biomarkers associated with each component are indicated. Evidence-based therapeutic targets and their components are shown at the bottom. CRP, C-reactive protein; IL-6, interleukin-6; TNF-α, tumor necrosis factor alpha; IGF-1, insulin-like growth factor 1; β-CTX, β-C-terminal telopeptide of type I collagen; PINP, N-terminal propeptide of type I procollagen; GDF-15, growth differentiation factor 15; IGFBP-2, insulin-like growth factor binding protein 2; FGF-23, fibroblast growth factor 23; PNI, Prognostic Nutritional Index; GNRI, Geriatric Nutritional Risk Index; CONUT, Controlling Nutritional Status; HALP, Hemoglobin, Albumin, Lymphocyte, and Platelet; AKI, acute kidney injury; SSI, surgical site infection; DVT, deep vein thrombosis; ADL, activities of daily living; ONS, oral nutritional supplements; BCAA, branched-chain amino acids; HMB, β-hydroxy-β-methylbutyrate; ERAS, Enhanced Recovery After Surgery; IHFCP, Integrated Hip Fracture Care Pathway; EHR, electronic health record; MDT, multidisciplinary team.

The pathophysiological underpinnings extend beyond mechanical load transmission. Muscle and bone function as active endocrine organs, communicating bidirectionally through paracrine and endocrine signaling via “myokines” and “osteokines,” and dysregulation of this intercellular dialogue drives osteosarcopenia pathogenesis (104). A large-scale circulating proteome association study involving over 6,000 participants identified growth hormone/IGF system proteins (growth hormone receptor, IGF binding protein 2), GDF-15, and EGFR as strongly associated with incident HF risk, alongside activation of acute-phase response signaling pathways (105). The acute catabolic state post-fracture exacerbates this process through accelerated muscle wasting and bone loss, which may be partially mitigated by early nutritional intervention (106). Fibrosis markers such as TGF-β1 demonstrate sex-specific associations with HF risk, which is protective in women but potentially deleterious in men with systemic inflammation, revealing the biological complexity underlying these biomarker associations (107). Figure 2 provides a comprehensive pathophysiological framework illustrating the malnutrition-inflammation-osteosarcopenia nexus, delineating the cascade from the acute catabolic response triggered by HF through the mutually reinforcing triad to adverse clinical outcomes and evidence-based therapeutic targets.

### 6.2 Novel biomarkers: from myostatin to bone turnover markers

Specific serum biomarkers are emerging as tools for sarcopenia assessment and monitoring. Myostatin, a negative regulator of muscle growth, has been proposed as a candidate sarcopenia biomarker (108). A preliminary study demonstrated significant myostatin reduction in HF patients undergoing rehabilitation with amino acid supplementation, with this decrease correlating with sarcopenia improvement (109). Vitamin D occupies a central integrative role for the reason that HF patients exhibit universally depressed vitamin D levels (4, 110), with male patients having significantly lower levels both at admission and 1-year post-fracture compared with females (111). Higher vitamin D concentrations correlate with better physical function, while lower vitamin D and higher bone resorption markers are associated with reduced bone density (111). In addition, Wu et al. (112) revealed that elevated serum β-CTX and PINP levels are associated with significantly increased 1-year mortality and walking disability risk, and their incorporation into predictive models substantially improves discriminatory performance.

To translate these findings into a robust clinical perspective, the underlying biological mechanisms of these emerging indicators must be delineated. Myostatin operates as a potent autocrine and paracrine inhibitor of muscle mass, binding to activin type IIB receptors to trigger the Smad2/3 signaling cascade, which suppresses protein synthesis and accelerates muscle proteolysis during the acute post-fracture catabolic phase. Concurrently, serum beta-CTX (a specific cleavage product of type I collagen degradation) and PINP (a byproduct of type I procollagen synthesis) provide a dynamic window into bone remodeling. The acute skeletal trauma decouples this balance, where pro-inflammatory cytokines heavily stimulate osteoclastic bone resorption (elevating beta-CTX) while temporarily blunting osteoblastic bone formation (altering PINP). Similarly, alterations in GDF-15 and the growth hormone/IGF system reflect a centralized metabolic shutdown, where systemic inflammation downregulates anabolic pathways, driving synchronous musculoskeletal decline.

Despite their physiological relevance, substantial barriers regarding analytical validity and reproducibility currently preclude these indicators from routine adoption. Unlike universally standardized assays for serum albumin, bone turnover markers (especially beta-CTX) are highly susceptible to profound diurnal variation, fasting status, and underlying renal clearance capacity, leading to significant cross-platform variability. Furthermore, commercial assays for serum myostatin and novel proteomic panels lack international reference standards, compromising their reproducibility across different institutional laboratories. Consequently, a strict distinction must be maintained. While classical protein indicators are fully clinically validated and actionable, these novel proteomic, bone, and muscle markers remain strictly exploratory tools that require rigorous multi-center assay standardization before they can be safely integrated into standard orthogeriatric care protocols.

### 6.3 From biomarkers to functional prediction

Beyond MNA-SF, which has been validated as the screening tool most closely associated with acute-phase functional recovery, emerging evidence emphasizes muscle quality over muscle quantity (27). Intramuscular fat infiltration and phase angle have been identified as superior short-term predictors of functional recovery compared with muscle mass alone (113). Ultrasound-measured muscle thickness (masseter, biceps, quadriceps) correlates with localized sarcopenia, malnutrition, and functional abilities including swallowing, self-feeding, and mobility (114). Even thigh muscle biopsy derived fiber diameter, together with mid-upper arm muscle circumference and serum albumin, has been identified as a direct risk factor for inability to walk independently at 6 weeks postoperatively (54). These findings all drive the assessment paradigm from macroscopic to microscopic, from static to dynamic, and from unidimensional to multidimensional evaluation.

## 7 From assessment to intervention: nutritional support strategies and outcomes

### 7.1 Oral nutritional supplements

In recent years, oral nutritional supplementation (ONS) represents the most extensively studied and readily implementable perioperative nutritional intervention. A meta-analysis of 18 randomized trials demonstrated that ONS significantly increased postoperative serum albumin levels and reduced infective complications by 46%, pressure ulcer incidence, and overall complication rates by approximately half, while also shortening LOS (115). Lai et al. (116) also concluded in their systematic review of preoperative ONS that protein-based supplements significantly reduced postoperative complications by 52%. Kim et al. (117) additionally found postoperative supplementation could attenuate the decline in serum protein and albumin levels, achieve higher albumin levels at 2-week follow-up, shorten LOS, and reduce delirium incidence.

### 7.2 Specific nutritional formulations and protocols

The composition and timing of nutritional interventions continue to be refined. In a propensity score–matched study, Kang et al. (118) concluded that branched-chain amino acid (BCAA) supplementation significantly improved postoperative serum albumin recovery. Pareja et al. (119) also demonstrated high-calorie, high-protein ONS including β-hydroxy-β-methylbutyrate shown good tolerability and compliance over 12 weeks, with significant improvements in nutritional status and biochemical parameters. Preoperative carbohydrate-whey protein beverages improved postoperative thirst and hunger, stabilized glucose fluctuations, elevated albumin levels, and reduced CRP concentrations (120). In a multicenter study, Liu et al. (121) demonstrated that Enhanced Recovery After Surgery (ERAS) protocols incorporating early nutrition showed efficacy in reducing postoperative complications by 33% and increasing home discharge rates by 24%.

### 7.3 Impact on laboratory indicators beyond classical nutritional markers

Nutritional interventions exert effects extending beyond classical protein markers. Individualized nutritional supplementation has been demonstrated to attenuate postoperative oxidative stress, evidenced by better control of advanced oxidation protein products and malondialdehyde levels and superior maintenance of total antioxidant capacity (122). High-energy intake regimens prevented the postoperative decline in IGF-1, a sensitive short-term nutritional marker, although they did not reverse the inflammation-mediated suppression of albumin and prealbumin in the early postoperative period (5). Vitamin D, which is profoundly deficient in the vast majority of HF patients, shows further significant decline in the early postoperative period, with this decline correlating with prolonged LOS (65). Serum vitamin E (α- and γ-tocopherol) concentrations correlate positively with post-fracture physical function recovery, suggesting this micronutrient as a potentially modifiable therapeutic target (123). These findings expand the biological objectives of nutritional intervention beyond energy-protein correction to encompass redox modulation and micronutrient optimization.

### 7.4 Functional outcomes and recovery trajectories

The ultimate objective of nutritional support is to promote functional recovery and improve long-term outcomes. In rehabilitation settings, nutritional improvement is independently associated with higher FIM scores at discharge (22). Nutrition trajectory analyses reveal that patients with poor nutritional trajectories exhibit worse self-care ability, quality of life, cognitive recovery, and greater depressive symptomatology (124). Critically, good nutritional trajectories can buffer the deleterious impact of cognitive impairment on ADL recovery, whereas coexistent malnutrition and cognitive impairment produce the worst functional outcomes (125). Social support trajectories interact with nutritional status that patients with high and stable social support demonstrate superior nutritional and physical function profiles (126). Multidisciplinary nutritional care has also been demonstrated to increase inpatient protein and energy intake and, at 3-month follow-up, to reduce the proportion of malnourished patients and attenuate quality-of-life decline (127). The current evidence for perioperative nutritional interventions is summarized in Table 4.

**Table 4:** Evidence summary for perioperative nutritional interventions in geriatric hip fracture patients.

Intervention	Study design	Key outcomes	Effect on laboratory indicators	Limitations/ Notes	References
ONS (general, postoperative)	Meta-analysis of 18 RCTs	46% reduction in infective complications (OR 0.54); reduced pressure ulcers; reduced LOS	Increased albumin (WMD 1.24 g/L)	Compliance 64.7%−100%; no consistent mortality benefit	(115)
Preoperative protein-based ONS	SR/MA of 5 RCTs	52% reduction in postoperative complications (OR 0.48)	Not specifically reported	No effect on LOS or mortality; limited preoperative evidence	(116)
Postoperative ONS (THA patients)	Prospective controlled	Reduced LOS; reduced delirium incidence	Attenuated albumin decline; higher albumin at 2-week follow-up	Single surgical population (THA)	(117)
BCAA supplementation	PSM study	Improved postoperative albumin recovery	Significant increase in serum albumin	Observational design; requires RCT validation	(118)
High-calorie/high-protein ONS with HMB	Prospective observational (12 weeks)	Improved nutritional status and biochemical parameters	Improved nutritional biochemical markers	Good tolerability and compliance	(119)
Preoperative CHO-whey protein drink	Interventional	Reduced thirst/hunger; stabilized blood glucose	Increased albumin; decreased CRP	Improves symptomatic and metabolic recovery	(120)
ERAS protocols	Multicenter study	33% reduction in complications (RR 0.67); 24% increase in home discharge (RR 1.24)	Multicomponent; nutritional effect not isolated	Early nutrition is one component of comprehensive protocol	(121)
MDT-led nutritional care	Prospective controlled cohort	Increased 24-hour energy intake (2,957 → 6,224 kJ); increased community discharge (48.0 vs. 17.6%)	Reduced nutritional deterioration (5.4 vs. 20.5%)	Multidisciplinary, multi-modal model compared against conventional individualized dietitian-led care; before-after design	(153)

ONS, oral nutritional supplements; MA, meta-analysis; SR, systematic review; RCT, randomized controlled trial; PSM, propensity score matching; BCAA, branched-chain amino acids; HMB, β-hydroxy-β-methylbutyrate; CHO, carbohydrate; ERAS, Enhanced Recovery After Surgery; MDT, multidisciplinary team; THA, total hip arthroplasty; LOS, length of stay; WMD, weighted mean difference; CRP, C-reactive protein; OR, odds ratio; RR, rate ratio.

### 7.5 Controversies and implementation challenges

Despite the overall positive evidence, important controversies and barriers remain. Several studies have failed to demonstrate significant effects of ONS on mortality, readmission, or certain perioperative complications (128–130). Ashkenazi et al. (130) found that although ONS significantly increased discharge albumin levels, it did not reduce complications or mortality. These discrepancies likely reflect heterogeneity in study design, intervention timing, duration, baseline nutritional status, and compliance (115). Preoperative supplementation faces logistical challenges related to surgical urgency, and evidence remains relatively sparse (131). Post-discharge continuation for at least 2–3 months appears necessary to meet nutritional requirements (132, 133), and cost-effectiveness analyses yield equivocal results depending on outcome measures used (134).

These controversies are further complicated by implementation barriers. Dixon et al. (23) in their research revealed that 24.2% received no oral intake preoperatively, with 6.34% fasted for over 36 h, despite 15.3% of nil-by-mouth patients being at nutritional risk. Similarly, in a large retrospective study of 160,151 patients, Williams et al. (135) found only 4.9% of malnourished patients received early postoperative nutritional supplementation, indicating profound underutilization. Moreover, Beric et al. (136) revealed that dysphagia affecting 54% of HF patients postoperatively, further limits oral nutritional interventions. However, educational interventions for healthcare staff have shown promise. Xie et al. (137) recently reported that nutrition knowledge scores increased from 61.07 to 85.57 after training, leading to improved protocol adherence. These data all highlight the critical need for systemic changes encompassing standardized protocols, staff education, and patient-centered care to bridge the gap between evidence and practice.

## 8 Multidisciplinary integration: applications and challenges

### 8.1 Integrating laboratory indicators into multidisciplinary frameworks

The reconceptualization of HF as a systemic disease event necessitates management strategies that transcend isolated orthopedic repair, embracing comprehensive multidisciplinary team approaches involving orthopedic surgeons, geriatricians, rehabilitation physicians, physical therapists, dietitians, nurses, and pharmacists (1, 11). In this framework, nutrition-related laboratory markers function as an objective, quantifiable universal language that connects diverse disciplines and guides personalized treatment decisions.

First, nomogram models, as preoperative risk stratification tools, integrating age, Charlson Comorbidity Index, serum albumin, sodium, and hemoglobin have achieved C-indices of 0.76 for predicting postoperative mortality (14). CONUT and PNI scores independently predict walking ability loss and long-term ADL dependence (82, 85), while perioperative troponin elevation signals heightened mortality and cardiac complication risk (138). Second, these indicators guide targeted complication prevention by providing the MDT with evidence-based triggers for enhanced anticoagulation, volume optimization, nutritional support, and delirium prevention. Third, laboratory indicators can serve as intervention monitoring tools. Nutritional support demonstrably improves albumin and prealbumin levels and reduces delirium incidence (5, 117), while home-based intervention programs have been shown to reduce mortality and improve mobility (139).

Integrated Hip Fracture Care Pathways (IHFCPs), incorporating early nutritional screening and dietitian consultations, have been associated with significant reductions in time to surgery and LOS (140). Fracture liaison services expanded to include nutritional assessment have increased osteoporosis treatment rates to 65% (141). In a small single-center quality-improvement study, embedding a clinical dietitian within an orthopedic ward, supported by iterative Plan-Do-Study-Act cycles, increased the proportion of at-risk patients meeting their energy and protein requirements from 22% to 80% and increased documented nutrition-risk screening from 10 to 80%, though the sample size was small (142). MDT co-management models have also been associated with reduced time to surgery and LOS (143). These organizational innovations demonstrate the transformative potential of systematic nutritional integration within multidisciplinary frameworks.

### 8.2 Challenges in clinical translation

Several challenges impede optimal integration. First, markers such as albumin and CRP are jointly influenced by nutrition and inflammation, making it difficult to disentangle their relative contributions during the acute post-fracture period (5, 91). Some promising novel biomarkers, such as soluble urokinase plasminogen activator receptor, have shown limited predictive value in high-risk HF patients compared with traditional indicators (144). Second, preoperative and early postoperative values may carry different prognostic implications: early postoperative hypoalbuminemia predicts AKI more reliably than preoperative creatinine (47, 100), and POD risk relates to both preoperative inflammatory and nutritional status (87, 97). This necessitates structured monitoring protocols at defined perioperative time points. Third, evidence-to-practice gaps persist. While abundant evidence links multiple indicators to outcomes, the precise multidisciplinary intervention “bundle” to trigger when PNI or GNRI falls below threshold, remains incompletely defined (133). Fourth, orthopedic surgeons may focus on hemoglobin and D-dimer, geriatricians on albumin and inflammatory indices, and rehabilitation teams on functional correlates of nutritional markers (11, 145). Without standardized, consensus-based interpretation and response protocols, interventions may remain fragmented. Finally, resource constraints and practical considerations, including the complexity of machine learning models requiring external validation (89) and the development of simplified assessment tools such as the Fracture Mobility Score (146) influence the feasibility of implementing comprehensive monitoring in real-world settings.

### 8.3 Clinical utility assessment: incremental discrimination, interventional efficacy, and implementation readiness

Biomarkers must demonstrate added discriminative power over established clinical risk scores to justify routine use. Many novel composite ratios such as GAR, CAR, DAR are currently evaluated only in isolation, lacking rigorous reclassification or head-to-head validation against existing clinical frameworks. While baseline biomarker depletion strongly correlates with adverse prognosis, evidence for true biomarker-guided, threshold-driven therapy remains limited. Universal perioperative ONS demonstrates clear efficacy, elevating postoperative albumin, reducing infective complications, and shortening LOS. Specialized protocols, such as BCAA supplementation, also accelerate postoperative protein recovery. However, a major evidence-to-practice gap persists that current protocols apply nutritional support universally rather than implementing biomarker-triggered adaptive care pathways. Prospective validation of specific threshold-driven intervention bundles is critical for clinical translation.

To guide clinical practitioners, nutrition-related indicators are stratified by their current operational maturity. Serum albumin and prealbumin are fully validated, standardized, and cost-effective for baseline screening and dynamic perioperative monitoring. Composite scores including PNI, GNRI, and CONUT rely entirely on routine panels (albumin, cholesterol, lymphocytes) and can be immediately embedded into electronic health record alert systems without adding institutional costs. Remaining in the Investigational Phase: Novel circulating proteomic markers like GDF-15, EGFR, and IGF system proteins offer robust long-term prognostic value but lack assay standardization and affordability. Specific sarcopenia markers (myostatin) and bone turnover markers (PINP) provide granular musculoskeletal insights but lack consensus regarding therapeutic thresholds.

### 8.4 Future directions for intelligent management pathways

The path forward lies in constructing intelligent, dynamic management pathways. Nomograms and electronic health record–integrated systems that automatically stratify risk and trigger alerts based on multidimensional data including laboratory indices, comorbidities, and functional assessments, can facilitate rapid identification of high-risk patients (14, 102, 147). Novel composite indices, such as the lymphocyte ratio-calcium index (LRCa3), may more precisely capture pathophysiological processes linked to functional recovery (148). The integration of point-of-care testing for biomarkers such as IGF-1 and CRP enables real-time nutritional assessment and timely intervention (5). Ultimately, these advances must converge upon establishing laboratory-indicator–triggered precision response mechanisms by linking specific threshold abnormalities to standardized multidisciplinary intervention packages, including intensive nutritional support, individualized rehabilitation, pharmacological optimization, and targeted complication prophylaxis, with continuous monitoring to evaluate effectiveness and dynamically adjust management. Only through such deep integration of biological data into clinical decision-making can the field progress from the current paradigm of generic management toward truly patient-centered, evidence-driven precision care.

## 9 Critical appraisal of evidence quality and methodological limitations

### 9.1 Distribution of evidence levels and retrospective design vulnerabilities

A rigorous translation of nutrition-related clinical laboratory indicators into bedside practice demands a critical appraisal of the architectural quality of the underlying evidence. Currently, the vast majority of available data linking biomarkers to geriatric hip fracture outcomes are derived from retrospective, observational cohort designs, representing Level III or Level IV evidence. While large-scale retrospective studies provide immense statistical power to detect independent associations, they possess inherent methodological vulnerabilities. These include susceptibility to selection bias, historical variance in surgical and perioperative care protocols, and a substantial risk of missing data, particularly regarding the dynamic tracking of biomarkers post-admission. The reliance on retrospective data introduces an unavoidable risk of bias, as the timing of blood draws, fluid resuscitation volumes, and the initiation of nutritional support are rarely standardized in retrospective cohorts, unlike in rigidly controlled prospective trials.

### 9.2 The inflammation-nutritional confounding nexus and biomarker misinterpretation

A pivotal limitation in the current literature is the profound confounding effect of systemic inflammation on classical protein nutritional indicators. Throughout the existing literature, serum albumin and prealbumin are frequently presented as pure surrogates for baseline nutritional status or somatic protein reserves. However, from a pathophysiological standpoint, both molecules are negative acute-phase reactants. The acute skeletal trauma of a hip fracture, combined with the subsequent stress of major orthopedic surgery, triggers a massive systemic inflammatory cascade characterized by the hyper-secretion of pro-inflammatory cytokines such as IL-6 and TNF-α. This hyper-inflammatory state profoundly suppresses hepatic synthetic pathways, favoring the production of positive acute-phase reactants (e.g., C-reactive protein) at the direct expense of albumin and prealbumin. Furthermore, inflammation dramatically increases capillary permeability, leading to the transcapillary escape of albumin into the interstitial space. Consequently, a precipitously dropping perioperative albumin level may reflect the magnitude of the surgical stress response and fluid shift volume expansion rather than acute nutritional starvation. Beyond inflammation, these biochemical signals are heavily confounded by baseline frailty, advanced age, and pre-existing comorbidity burdens such as chronic kidney disease or hepatic impairment, which independently alter biomarker concentrations and mask the true nutritional signal.

### 9.3 Statistical association vs. biological causality

Clinicians and researchers must carefully distinguish between statistical association and biological causality when interpreting biomarker data in this vulnerable population. The correlations presented in current clinical literature that linking hypoalbuminemia or depressed PNI values to postoperative pneumonia, delirium, acute kidney injury, and long-term mortality, establishes a robust prognostic association, but does not definitively prove a causal nexus. For instance, it remains heavily debated whether a low preoperative albumin level actively drives the pathogenesis of postoperative pneumonia via immune impairment, or whether it merely serves as a non-specific proxy indicator for a profoundly frail patient with a collapsed physiological reserve who is inherently predisposed to pulmonary collapse. Because malnutrition, systemic inflammation, frailty, and sarcopenia operate as a tight, mutually reinforcing pathophysiological loop (the osteosarcopenia-nutrition nexus), disentangling independent causal pathways via observational data is methodologically unfeasible. This highlighted ambiguity underscores the critical need for future multi-center prospective studies and randomized controlled trials that utilize advanced multivariate structural equation modeling and causal inference frameworks to evaluate whether targeted correction of these specific laboratory thresholds directly prevents subsequent organ complications.

## 10 Conclusions and future perspectives

This review synthesizes a comprehensive body of evidence establishing the central prognostic and clinical utility of nutrition-related laboratory indicators in the perioperative management of geriatric HFs. Classical protein markers including serum albumin and prealbumin, serve as robust, independent predictors of postoperative complications, functional recovery failure, and short- and long-term mortality (19, 41, 57, 58, 62). Inflammatory-nutritional composite indices such as PNI, GNRI, CONUT, CAR, GAR, and SII, provide enhanced risk stratification by capturing the pathological convergence of inflammation and nutritional depletion (26, 30, 33, 73, 78, 86). These readily accessible biomarkers constitute the objective, quantifiable foundation upon which precision perioperative management can be constructed.

**Figure 3:**
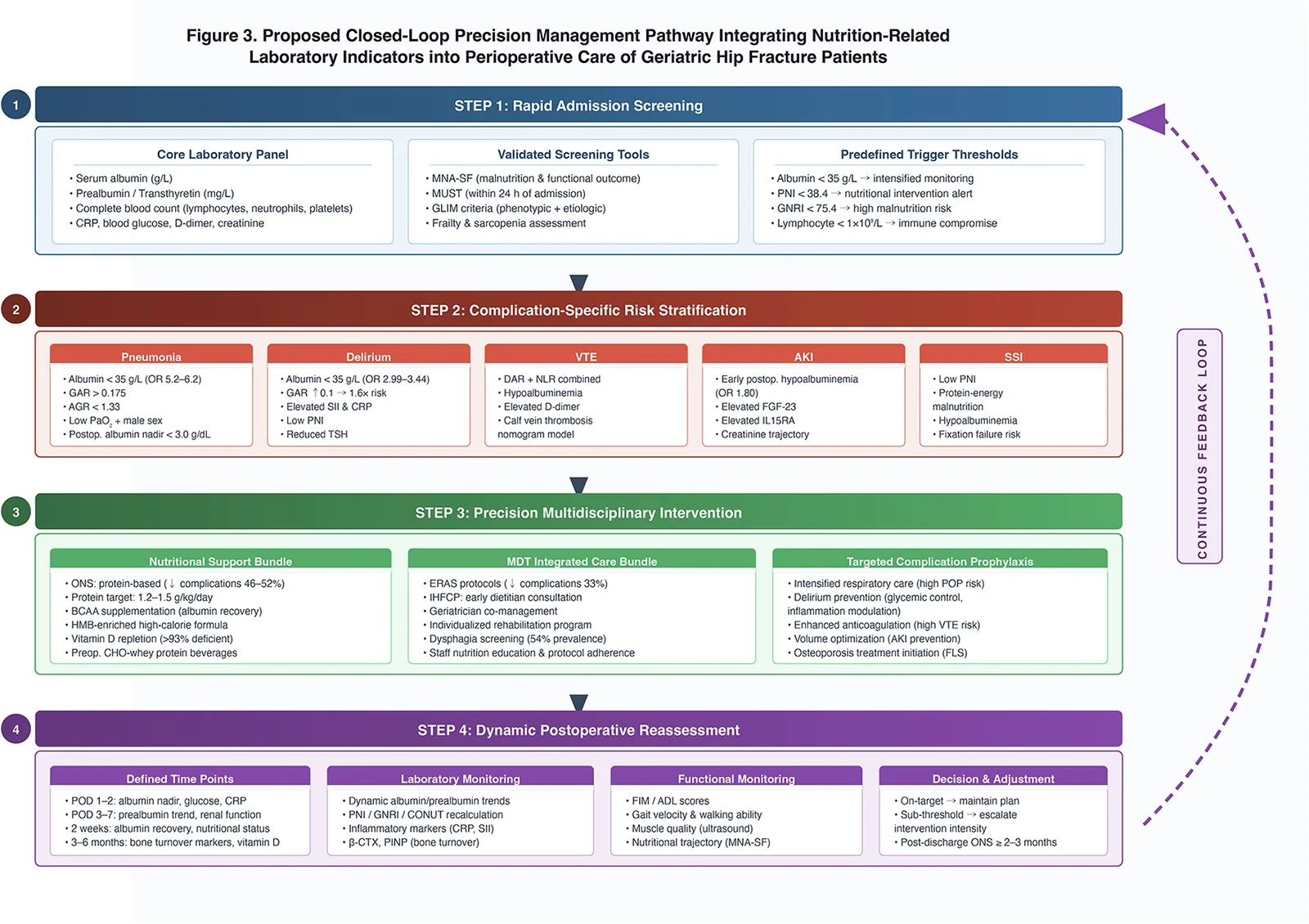
Proposed closed-loop precision management pathway integrating nutrition-related laboratory indicators into perioperative care of geriatric hip fracture patients. The pathway comprises four sequential steps: (Step 1) rapid admission screening using a core laboratory panel and validated tools with predefined trigger thresholds; (Step 2) complication-specific risk stratification deploying targeted biomarker combinations for pneumonia, delirium, venous thromboembolism, acute kidney injury, and surgical site infection; (Step 3) precision multidisciplinary intervention guided by identified risk profiles, encompassing nutritional support and integrated care bundles; and (Step 4) dynamic postoperative reassessment at defined time points with laboratory and functional monitoring. The closed-loop arrow **(right)** indicates that reassessment findings continuously feed back into re-stratification and intervention modification. Note this proposed clinical decision-making pathway and its specified indicator thresholds are currently conceptual and hypothetical, serving as a framework for future research that strictly mandates rigorous prospective validation in clinical settings. CRP, C-reactive protein; MNA-SF, Mini Nutritional Assessment Short-Form; MUST, Malnutrition Universal Screening Tool; GLIM, Global Leadership Initiative on Malnutrition; PNI, Prognostic Nutritional Index; GNRI, Geriatric Nutritional Risk Index; GAR, glucose-to-albumin ratio; AGR, albumin-to-globulin ratio; SII, Systemic Immune-Inflammation Index; CRP, C-reactive protein; PNI, Prognostic Nutritional Index; DAR, D-dimer-to-albumin ratio; NLR, neutrophil-to-lymphocyte ratio; FGF-23, fibroblast growth factor 23; IL15RA, interleukin-15 receptor alpha; ONS, oral nutritional supplementation; BCAA, branched-chain amino acid; HMB, β-hydroxy-β-methylbutyrate; ERAS, Enhanced Recovery After Surgery; IHFCP, Integrated Hip Fracture Care Pathway; POP, postoperative pneumonia; AKI, acute kidney injury; POD, postoperative delirium; CONUT, Controlling Nutritional Status; β-CTX, β-C-terminal telopeptide of type I collagen; PINP, N-terminal propeptide of type I procollagen; ADL, activities of daily living; FIM, Functional Independence Measure.

The future management paradigm should be conceptualized as a closed-loop system: screening-risk stratification-precision intervention-reassessment. A streamlined core indicator panel comprising admission albumin, prealbumin, PNI, GNRI, or CONUT, should serve for initial rapid screening, with values below validated thresholds automatically triggering intensified assessment and monitoring (30, 62, 81). Complication-specific prediction should deploy targeted indicator combinations. For example, GAR with cognitive assessment for delirium risk (72); DAR combined with NLR for venous thromboembolism risk (76); and low albumin with hypoxemia for pneumonia risk (93). Figure 3 operationalizes this paradigm into a proposed closed-loop precision management pathway, detailing the four sequential steps of rapid admission screening, complication-specific risk stratification, precision multidisciplinary intervention, and dynamic postoperative reassessment with continuous feedback-driven modification. However, it must be explicitly emphasized that many of the proposed biomarker thresholds and intervention algorithms within this framework remain largely hypothetical and are synthesized predominantly from observational data. Thus, they strictly require robust, prospective clinical validation through multi-center trials before widespread bedside implementation.

### 10.1 Prioritized future research agenda

Despite the substantial body of evidence synthesized in this review, most nutrition-related laboratory indicators for geriatric HF have been derived from single-center, retrospective, or observational studies with heterogeneous cut-off values, patient selection, and outcome definitions. To translate these promising biomarkers into standardized precision-medicine tools, we propose the following four hierarchically prioritized research directions:

(1) Prospective multicenter validation studies (Priority 1-highest immediate need). Large-scale prospective, multicenter cohort studies are urgently required to externally validate the prognostic performance of both single biomarkers (e.g., serum albumin, prealbumin) and composite indices (PNI, GNRI, CONUT, CAR, GAR, SII, HALP) across geographically, ethnically, and clinically diverse geriatric HF populations. Such studies should adopt harmonized data-collection protocols, standardized outcome definitions (e.g., 30-day, 90-day, and 1-year mortality; Clavien–Dindo-graded complications; validated functional recovery instruments), and pre-specified analytical plans to enable rigorous meta-analytic synthesis and generalizable clinical translation (26, 30, 62).
(2) Standardization and harmonization of biomarker thresholds (Priority 2-foundational). The current literature is characterized by markedly heterogeneous cut-off values for the same biomarker across studies (e.g., PNI thresholds ranging from 38 to 47; albumin thresholds from 30 to 38 g/L), which severely limits comparability and clinical adoption. International consensus efforts, informed by pooled individual patient data meta-analyses and receiver-operating-characteristic modeling stratified by age, sex, fracture type, and comorbidity burden, are needed to establish validated, universally applicable cut-off thresholds, together with clear reporting standards analogous to STARD and TRIPOD frameworks (19, 41, 57).
(3) Formal integration with the GLIM criteria (Priority 3-diagnostic convergence). Nutrition-related laboratory biomarkers should be systematically embedded within, and prospectively benchmarked against, the GLIM diagnostic framework, which currently emphasizes phenotypic and etiologic criteria but incompletely operationalizes inflammatory and biochemical dimensions (34). Prospective studies are required to determine (i) whether composite indices such as CONUT, PNI, or CAR can serve as validated etiologic-criterion surrogates for disease burden/inflammation, (ii) whether their addition improves the sensitivity and prognostic accuracy of the GLIM algorithm in geriatric HF, and (iii) how biomarker-augmented GLIM diagnosis maps onto downstream intervention decisions.
(4) Randomized controlled trials of biomarker-guided nutritional interventions (Priority 4-translational endpoint). The pivotal evidence gap between prognostic biomarker performance and clinical benefit must be closed through adequately powered randomized controlled trials in which nutritional interventions (e.g., high-protein oral nutritional supplementation, immune-nutrition, branched-chain amino acid supplementation, ERAS-embedded protocols) are triggered, titrated, and dynamically adjusted according to specific laboratory-indicator thresholds, and are compared against usual perioperative care. Such trials should employ pragmatic multicenter designs, patient-centered composite outcomes (survival, complication burden, functional recovery, quality of life, and readmission), and health-economic evaluations to demonstrate whether biomarker-guided precision nutrition delivers superior, cost-effective outcomes than standard practice (124, 131, 152).

Complementing these four core priorities, several forward-looking horizons will further shape the field: the discovery and validation of novel biomarkers specifically predictive of functional recovery trajectories, exemplified by emerging indices such as LRCa3 (148) and large-scale proteomic studies identifying GDF-15 and IGF-system proteins (105, 149); the application of machine learning and artificial intelligence to integrate laboratory indicators with clinical, imaging (e.g., CT-derived bone density indices) (150), and multi-omics data for individualized prognostic modeling; and the development of digital monitoring platforms (142), genetically and metabolically personalized nutrition strategies (151), and integrated exercise–nutrition intervention paradigms (152).

Closing these prioritized evidence gaps through prospective multicenter validation, threshold standardization, GLIM integration, and biomarker-guided interventional trials constitutes the critical translational pathway from the current, predominantly observational biomarker literature to genuine precision perioperative care. The ultimate objective is to convert the accumulated wealth of biomarker evidence into actionable, standardized, and internationally harmonized clinical decision pathways that seamlessly embed within multidisciplinary collaborative care. By anchoring perioperative decision-making in objective laboratory data and by establishing rigorously validated linkages between biomarker thresholds and specific, evidence-supported intervention bundles, the field can advance from generic perioperative management toward true precision medicine for this profoundly vulnerable population, ultimately improving survival, restoring functional independence, and reducing the global socioeconomic burden of geriatric HF.
